# Current Diagnostic Status of Pheochromocytomaand Future Perspective: A Mini Review

**Published:** 2017-07-01

**Authors:** Fatemeh Khatami, Seyed Mohammad Tavangar

**Affiliations:** 1 *Chronic Diseases Research Center, Endocrinology and Metabolism Population Sciences Institute, Tehran University of Medical Sciences, Tehran, Iran*; 2 *Dept. of Pathology, Dr. Shariati Hospital, Tehran University of Medical Sciences, Tehran, Iran*

**Keywords:** Pheochromocytomas(PCCs), Histopathology, Genes, Catecholamines, Metanephrines

## Abstract

Pheochromocytomas (PCCs) are rare neuroendocrine tumors. The current diagnostic tools are based on biochemistry and histopathology results, but heterogeneity of diagnostic markers, signs and symptoms of PCCs bring a lot of difficulties for these two current methods. Unfortunately, microscopic understanding of PCCs is not adequate for its confident prognosis and management. There are data linking specific genotypes of PCCs tumors to specific locations, typical biochemical phenotypes or future clinical behaviors. The detection of a germ-line mutation possibly can guide us to an early diagnosis, appropriate treatment, and regular surveillance with better prognosis for patients but also and their family members. Moreover, the latest discoveries in gene sequencing, circulating DNA (ctDNA) and circulating tumor cells (CTCs) will support the exact molecular pathogenesis of PCCs to provide an important basis for future PCCs managements.

## Introduction

Pheochromocytomas (PCCs) are infrequent, catecholamine-secreting, neuroendocrine tumors originated from chromafﬁn cells of the adrenal medulla (1). The annual incidence rate of pheochromocytoma is estimated around 0.8 per 100,000 person-years (2-4). Even though pheochromocytom may happen at any age, they are most widespread in the fourth to fifth decade (5). PCCs symptoms are diaphoresis, headache, sweating, palpitations, and cardiovascular problem such as myocardial infarctions, cardiomyopathy, and stroke (6). In spite of the fact that PCCs is associated with catecholamines increase in the circulation, some patients do not have any symptoms, so the diagnosis of PCCs can be more complicated (7). Most tumors can be recognized easily by determining some exact clinic-pathological features (8-12). Because of various clinical manifests of PCCs, timely and precise diagnosis is a problematic issue. In fact PCCs can happen at any age with equal distribution in male and female, however the age of patients may indicate to the tumor’s catecholamine phenotype and some fundamental genetic mutations (13, 14). Usually patients with an established mutation or hereditary syndrome could occur at a younger age than those with sporadic disease, whereas epinephrine secreting tumors happen at a later age (15). PCCs in elderly patients are typically sporadic and may present without classic signs and symptoms. Instead, some unexpected critical illness like stroke, heart failure in absence of coronary artery or vascular disease, and ketoacidosis can be seen (15, 16). Some symptoms of orthostatic hypotension such as lightheadedness, pre-syncope and syncope may be in patients with predominantly epinephrine or dopamine secreting tumors (17).

Microscopic histological characteristics of some endocrine tumors including PCCs are not satisfactory for exact benign and malignant tumors discrimination (18-20). As a result of this variety in clinical signs improvements in diagnosis, localization, management, and treatment of PCCs is extremely required to understanding its genetics and biology. The present position of microscopic pathology in management of PCCs is restricted to diagnosis and documentation of tumor behavior that may be clues to malignant potential (21). It is anticipated that future roles will involve more definitive assessment of malignancy using genetic and epigenetic biomarkers, genotype–phenotype correlation, and discovery of target genes for personalized PCCs therapy.

## Biochemical Diagnosis of Pheochromocytoma

Usually diagnosis of PCCs requires approval by several tests like biochemical evidence of excessive catecholamine production. The most common traditional test is urinary catecholamine levels, urinary catecholamine metabolites or plasma catecholamines measurements (22-25). Catecholamines are metabolised within chromaffin cells to metanephrines (i.e. norepinephrine to normetanephrine and epinephrine to metanephrine), and measurements of fractionated metanephrines in urine or plasma offer greater diagnostic sensitivity to measurements of the parent catecholamines (26-29). Analysis of 24 hour urinary metanephrines and normetanephrines offer the greatest sensitivity and specificity and negative plasma free metanephrines can be excluded for PCCs (29). It was shown that basal plasma catecholamine levels were elevated in 81 of 87 (93%) patients (30). Also, chromogranin-A concentrations in plasma have been found to be raised in PCCs patients (31), but its prognostic significance still under debate. Dopamine (DA) secretion has been suggested as a indicator of PCCs malignancy (32, 33). Moreover, increasing level of aromatic L-amino acid decarboxylase (ALAAD) has been shown frequently in malignant PCCs (30). The tumor can be localized anatomically by computed tomography (CT), magnetic resonance imaging (MRI) or Metaiodobenzylguanidine (MIBG) scanning after the diagnosis has been biochemically established. 

Extraordinarily, some PCCs could be "silent", because they may produce inadequate amounts of catecholamines and therefore no typical symptoms and signs and no positive biochemical tests (34). Moreover, PCCs produce catecholamines episodically, so plasma concentrations or urinary excretion of catecholamines will not be abnormal (35). Therefore, biochemical testing of plasma or urinary catecholamines and urinary metabolites of catecholamines are not constantly reliable to prove the presence of a PCCs tumor (36-38). A developed biochemical test that suggests measurements of plasma free normetanephrine and metanephrine, offered some advantages for biochemical testing of PCCs but it has some weak points as well (39, 40). 

## Current Roles of Pathology in Pheochromocytomas's Managements

Usually PCCs is a rounded, single mass with distortions of the gland that can be surrounded by the cortex if the tumor is small, whereas recognition of an adrenal remnant is not easy with extremely large tumors (41, 42). The diameter of PCC tumors generally is 3 to 5 cm with a wide range from 1 to 10 cm or more. The tumor is graywhite to tan, tight circumscribed, and usually encapsulated in cross-sectional view (43).Microscopically PCCs usually have a nesting or alveolar form with separate nests of cells ("zellballen"), or a mixture of both (44). The nuclei of the tumor cells possibly have pseudo inclusions, and also while the cytoplasm of PCCs cells stay often lightly basophilic and finely granular (45). The cytoplasm frequently has neurosecretory granules and infrequently melanin-like pigments (46, 47). In spite of the fact of weakness of histological features in exact determining of malignancy, there are some suggesting features that can be associated with malignancy like necrosis, vascular invasion, and extensive capsular invasion (44, 48-50). In 2002 the Adrenal Gland Scaled Score (PASS) was introduced by Thompson for discrimination between benign tumors from malignant ones (51). It contains high cellularity, cellular monotony, mitotic figures, extension into adipose tissue, vascular invasion, capsular invasion, profound nuclear pleomorphism, and nuclearhyperchromasia and so on (51). 

Immunohistochemical (IHC) staining procedures available in most pathology laboratories are able to provide the essential characteristic of endocrine tumors including PCCs (9, 18, 52-57). Adrenal medullary cells and their tumors display positivity for various neuroendocrine markers, such as chromogranin A, major proteins in the neurosecretory granules of neuroendocrine cells and sympathetic nerves, that has the highest concentration in the adrenal medulla. Chromogranin A (CgA), a major constituent of the matrix of catecholamine-containing secretary granules, is the single most specific and reliable generic neuroendocrine marker which is used in pathology practice (9, 58). Another IHC marker is a membrane glycoprotein of presynaptic vesicles, synaptophysin, which is reactive in neuronal presynaptic vesicles of the brain, spinal cord, retina, neuromuscular junctions and small vesicles of adrenal medulla and pancreatic islets (59). In fact, antibody to synaptophysin exactly stains neuronal, adrenal and neuroepithelial tumors, including adrenal and extra _ adrenal PCCs, pancreatic islet cell tumors, carcinoid tumors, pituitary/parathyroid adenomas, and thyroid medullary carcinoma (60, 61). There are some other suggesting neural tumors markers such as neuron-specific enolase (NSE) which is a glycolytic isoenzyme, the neural cell adhesion molecule (NCAM/CD56), S100 protein positive sustentacular cells, a melanoma marker Melan A (MART-1, A103)(62-66). 

## Genetics of Pheochromocytomas

It is a common knowledge that genetic and epigenetic alterations of tumors can provide evidence for superior tumor managements (67-71). According to the latest findings, around one-third of PCCs tumors are supposed to be triggered by germ-line mutations in at least 10 genes (72). These genes include: Rearranged during transfection (RET) proto-oncogene, Von Hippel–Lindau disease tumor-suppressor gene

(VHL), neurofibromatosis type 1 tumor-suppressor gene (NF 1), genes encoding four succinate dehydrogenase complex (SDH)(72-76),responsible enzyme for flavination of the SDHA subunit (SDH assembly factor 2 (SDHAF2)(77, 78), tumor-suppressor genes TMEM 127 and MAX(79-81). The specific linking genotypes of these genes and typical biochemical phenotypes or future clinical behavior have been suggested (82-84). On the other hand, clinical features, immunohistochemistry and catecholamine production could support the proper direction of genetic testing PCCs (85). More than these 10 genes there are some genes were reporting in connection with PCCs tumors, for example germ-line mutations of Kinsin family member 1B gene (KIF1B, located on chromosome 1p36.22)(86). KIF1B has a crucial role in cell response to hypoxia angiogenesis because it encodes a protein that provokes apoptosis (87). 

Regarding PCCs transcription profiles, two different groups of hereditary PCCs were identified including first cluster which has VHL and SDHx mutant tumors and the second cluster with RET and NF1 mutations (88, 89). VHL/SDHx transcription profile is linked to the angiogenesis, hypoxia and a reduced oxidative response through stabilizing HIF-a that is a transcription factor with important role in apoptosis, angiogenesis, energy metabolism, proliferation, migration and invasion of tumor cells(87, 88).RET/NF1 cluster cover genes concerned in initiation of translation, protein synthesis and kinase signaling(90) and is linked to the activation of the RAS/RAF/MAPK pathway and the PI3/AKT signaling pathway(87). Malignant PCCs is associated mostly with SDHB germ-line mutations (82, 83, 91). In fact, mutation of RET, VHL, NF1, TMEM-127 or MAX genes can suggest the intra-adrenal location of tumors (80, 92-94).

 And intra-adrenal tumors have been infrequently detected in germ-line mutations of SDHD, SDHA and SDHC (88, 94, 95). Bilateral PCCs have been typically associated with RET, VHL, TMEM-127 and MAX mutations (7, 96, 97). After diagnosis of extra-adrenal tumors, the germ-line mutations can be found mainly in SDHx genes (96, 98, 99). Extra-adrenal sympathetic tumors are generally linked to SDHB (particularly solitary, large tumors), less often to SDHD, rarely to SDHC and SDHA mutations (95, 100). If SDHD, SDHB and SDHC testing had negative results, then SDHAF2 mutation should be checked (101). In fact, discoveries in gene expression and the cellular pathways of PCCs will likely offer a basis for personalized medicine in near future. The presence of a germ-line mutation is possible in patients with every following feature; early onset (≤45 years), bilateral, multifocal or extra-adrenal tumors, recurrent or malignant disease and positive family history for PCCs(97, 102).Genetic testing of non-syndromic patients with apparently sporadic PCCs is dependent to histological evaluation, localization and catecholamine production (85). 

After the completion of human genome project, some epigenetic alteration of genome like methylation can be the key genetic changes leading to carcinogenesis in oncology field of study (103-105). Consequently epigenetics of PCCs can shed a light on its diagnosis and prognosis the same as other tumors (106). 

If we take the knowledge of liquid biopsy and modern advance molecular techniques into consideration, challenges for PCCs diagnosis, management and treatment could be solved (12, 71, 107, 108). Interestingly in the near future it will be easily possible to analyze all PCCs related genes in only one shot using the whole-genome sequencing (109).

## Conclusions

Despite several advances in pathology and microscopic measurements of PCCs, the precise management of this tumor has not been possible yet. There is a big opportunity that genetic and epigenetic of PCCs can provide additional data about PCCs. Therefore, various indicators for the exact genetic origin possibly will be checked in advance to improve biochemical and histology testing results. 
